# The emerging role of the Hippo signaling pathway in interorgan crosstalk

**DOI:** 10.1111/febs.70587

**Published:** 2026-05-14

**Authors:** Gahyeon Song, Chaerin Lee, Eek‐hoon Jho

**Affiliations:** ^1^ Department of Life Science University of Seoul Seoul Republic of Korea

**Keywords:** cancer progression, hippo signaling, interorgan communication, systemic homeostasis, YAP/TAZ

## Abstract

Interorgan communication has emerged as a fundamental mechanism for maintaining systemic homeostasis, and its disruption contributes to the development of metabolic diseases, chronic inflammation, and cancer. The Hippo signaling pathway, traditionally known for controlling organ size, has recently been redefined as a context‐dependent coordinator of systemic cues. By translating hormonal, metabolic, and microbial signals into coordinated cellular responses, Hippo signaling serves as a bidirectional communication hub that not only interprets systemic inputs but also generates “outputs” that connect multiple organ systems. In this review, we summarize how Hippo signaling integrates diverse stimuli within key interorgan axes (e.g., gut–liver, gut–pancreas, and adipose–peripheral organs), and how organ‐specific Hippo activity, particularly in adipose tissue and skeletal muscle, generates systemic outputs influencing metabolism. This integration occurs through context‐specific mechanisms, allowing Hippo signaling to adapt cellular programs to physiological or pathological conditions. Despite recent progress, the mechanisms by which Hippo signaling prioritizes and integrates multiple systemic inputs and transmits its effects across organs remain incompletely understood. Elucidating these processes will be crucial to establishing Hippo signaling as a unifying framework of interorgan communication and understanding its broader implications in systemic physiology and disease.

## Graphical abstract

Hippo signaling functions as a central hub of interorgan communication. Systemic cues from the gut, adipose tissue, and skeletal muscle—including hormones, metabolites, and microbial signals—regulate YAP/TAZ activity in a tissue‐ and context‐dependent manner. Through these mechanisms, Hippo signaling integrates organ‐derived stimuli to coordinate systemic homeostasis and pathological outcomes.

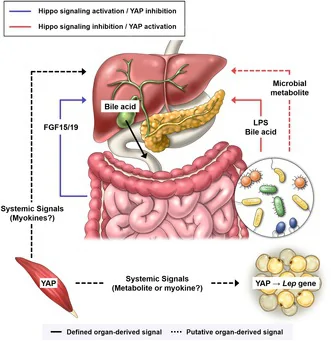

**Abbreviations**
5‐FU: 5‐fluorouracil
ABC: ATP‐binding cassette
ABX: antibiotic‐treated
BAIBA: β‐aminoisobutyric acid
CRC: colorectal cancer
EPAC: exchange protein directly activated by cAMP
ESCC: esophageal squamous cell carcinoma
FGF: fibroblast growth factor
FGF15/19: fibroblast growth factor 15/19
FMT: fecal microbiota transplantation
FXR: farnesoid X receptor
GLP‐1: glucagon‐like peptide‐1
GRP78: glucose‐regulated protein 78
HCC: hepatocellular carcinoma
HSPGs: heparin/heparan sulfate proteoglycans
IDH2: isocitrate dehydrogenase 2
IQGAP1: IQ motif‐containing GTPase‐activating protein 1
JAK/STAT: janus kinase/signal transducer and activator of transcription
LATS1/2: large tumor suppressor kinases 1/2
LBD: ligand‐binding domain
LPS: lipopolysaccharide
LSECs: liver sinusoidal endothelial cells
MAMPs: microbial‐associated molecular patterns
MAPK: mitogen‐activated protein kinase
MASH: metabolic dysfunction‐associated steatohepatitis
MASLD: metabolic dysfunction‐associated steatotic liver disease
Metrnl: meteorin‐like
MST1/2: mammalian STE20‐like 1/2
NAFLD: non‐alcoholic fatty liver disease
NASH: non‐alcoholic steatohepatitis
PanIN: pancreatic intraepithelial neoplasia
PCN: pregnenolone 16α‐carbonitrile
PDAC: pancreatic ductal adenocarcinoma
P‐gp: P‐glycoprotein
PI3K: phosphatidylinositol 3‐kinase
PKA: protein kinase A
PLCγ: phospholipase C gamma
PPARγ: peroxisome proliferator‐activated receptor‐γ
PXR: pregnane X receptor
RIF: rifampicin
SCFAs: short‐chain fatty acids
SHP: small heterodimer partner
TAZ: transcriptional coactivator with PDZ‐binding motif
TCA: tricarboxylic acid
TEAD: TEA domain transcription factor
TLR4: toll‐like receptor 4
Tregs: regulatory T‐cells
UCP1: uncoupling protein 1
VSMC: vascular smooth muscle cell
WAT: white adipose tissue
YAP: yes‐associated protein

## Introduction

Interorgan communication is essential for maintaining systemic homeostasis [1]. Individual organs do not function independently; instead, they are interconnected through complex, bidirectional networks mediated by circulating messengers, such as hormones, metabolites, and cytokines [2, 3, 4]. Under normal conditions, these communication networks coordinate systemic metabolism and immune responses; however, their dysregulation causes metabolic diseases, chronic inflammation, and cancer [5, 6, 7, 8]. Therefore, understanding how these interorgan communications are established and maintained at the molecular level is fundamental for elucidating both physiological homeostasis and disease development.

Multiple signaling pathways mediate organ–to–organ communication. For instance, the JAK/STAT pathway serves as a direct route for cytokines and hormones. The adipose‐derived hormone leptin acts on the hypothalamus through this pathway to regulate appetite and energy expenditure [9]. Another signaling route is the cAMP‐dependent pathway, through which gut‐derived hormones such as GLP‐1 regulate appetite and metabolic responses primarily via PKA and EPAC, with additional downstream engagement of PI3K/Akt signaling [10]. In addition, the MAPK pathway mediates adipose tissue browning induced by exercise‐induced myokines, such as irisin [11]. Although these pathways regulate specific physiological axes, the integration and interpretation of multiple organ‐derived cues at the systemic level remain poorly understood. Elucidating the molecular mechanisms that translate these interorgan signals into physiological outcomes is essential for understanding how systemic homeostasis is maintained and how its disruption contributes to disease.

**Fig. 1:**
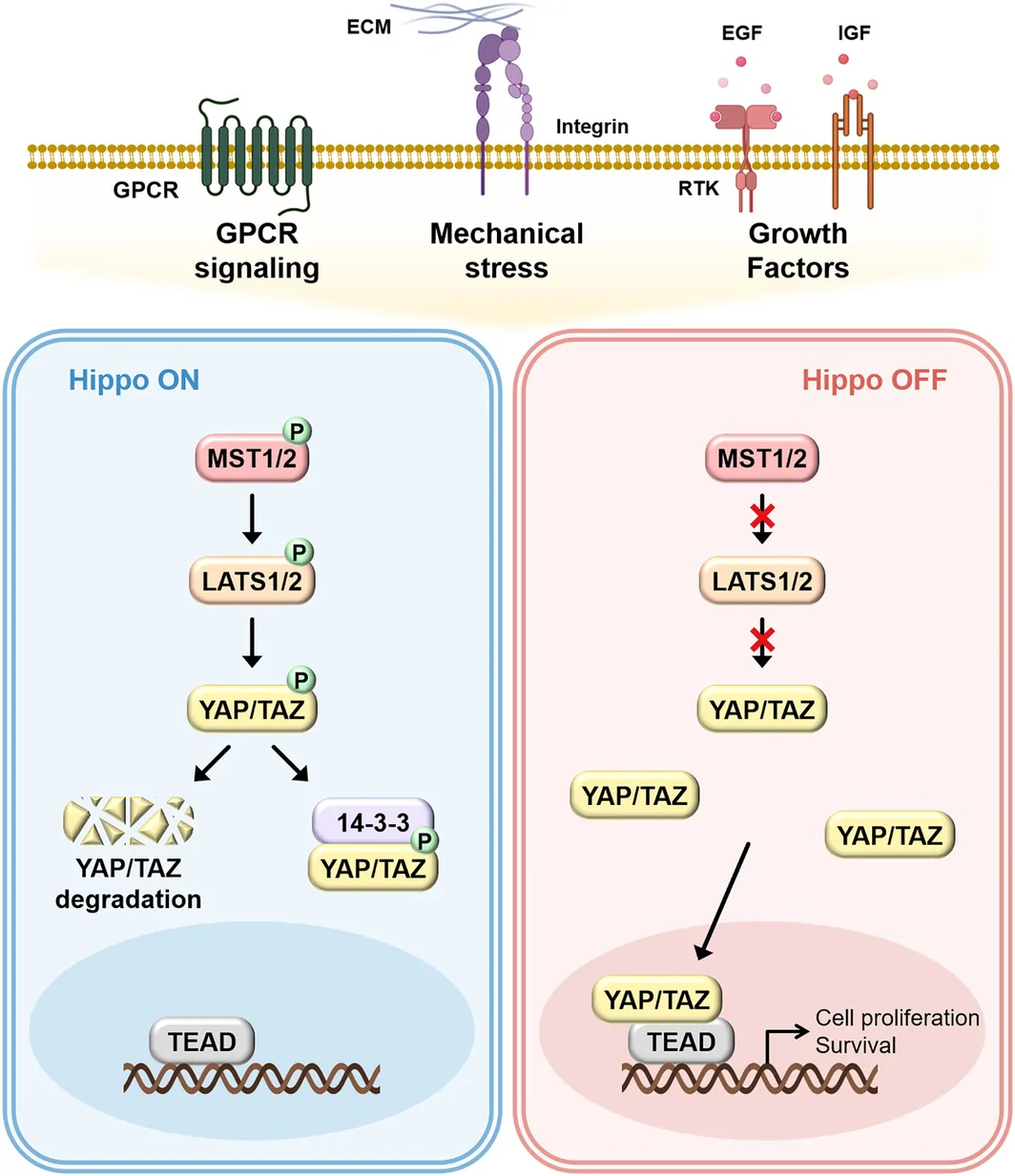
Canonical Hippo signaling and representative upstream inputs. Hippo signaling is regulated by diverse upstream cues, including GPCR signaling, mechanical stress, and growth factor receptor tyrosine kinase (RTK) signaling (e.g., EGF and IGF). When Hippo signaling is active (Hippo ON), MST1/2 phosphorylate and activate LATS1/2, which in turn phosphorylate YAP/TAZ, promoting 14‐3‐3 binding, cytoplasmic retention, and proteasomal degradation. When Hippo signaling is inactive (Hippo OFF), LATS1/2 fail to phosphorylate YAP/TAZ, allowing unphosphorylated YAP/TAZ to translocate into the nucleus, where they bind to TEAD family transcription factors and drive expression of genes involved in cell proliferation and survival.

The Hippo signaling pathway, originally identified as a master regulator of organ size, has since emerged as a multifaceted signaling network involved in tissue regeneration, metabolism, and tumorigenesis [12, 13, 14]. Canonically, Hippo signaling consists of a kinase cascade in which mammalian STE20‐like 1/2 (MST1/2) phosphorylates and activates large tumor suppressor kinases 1/2 (LATS1/2) [15, 16, 17]. Activated LATS1/2 then phosphorylate the transcriptional coactivators yes‐associated protein (YAP) and transcriptional coactivator with PDZ‐binding motif (TAZ), resulting in their cytoplasmic retention and degradation [18, 19]. When Hippo signaling is inactive, unphosphorylated YAP/TAZ enter the nucleus, where they bind to TEA domain transcription factor (TEAD) to induce gene expression related to cell proliferation and survival [12, 20] (Fig. 1, Table 1).

**Table 1:** Conventional versus expanded views of YAP/TAZ regulation: representative mechanisms discussed in this review.

	How YAP/TAZ is regulated	Representative examples
Conventional view	MST1/2‐LATS1/2 kinase cascade, which drives phosphorylation‐dependent inhibition	Cell–cell contact, polarity/junctional integrity, and cytoskeletal or mechanical cues that converge on the Hippo kinase cascade
Expanded view	Both canonical kinase‐cascade and non‐canonical mechanisms in response to interorgan cues	‐ Canonical kinase‐cascade regulation · FGF15/19‐FGFR4 signaling activates MST1/2‐LATS1/2 to suppress YAP in liver [46] (Fig. 2) ‐ Non‐canonical regulation · Bile acids activate YAP via a pathway dependent on the induction of the scaffold protein IQGAP1 [147] · PXR activates YAP independently of MST1/2‐LATS1/2 through deacetylation/ubiquitination in liver [61] (Fig. 2) · Gut‐derived bile acid/LPS signaling stabilizes YAP through autophagy inhibition in PDAC [82] (Fig. 3) · Leptin inhibits the AMPK‐LATS1 axis to activate YAP [97, 102] (Fig. 4A and B)

While Hippo signaling has been regarded as a cell‐intrinsic growth regulator [12, 16, 17, 21], recent evidence suggests that it may play more crucial roles under physiological stress—such as tissue regeneration, metabolic stress, or tumorigenesis—than in normal organ growth control [22, 23]. This paradigm shift raises a fundamental question: if Hippo signaling is not simply a universal growth controller, what is its core physiological function?

We suggest that the Hippo pathway acts as a sensor and interpreter of systemic signals, translating organ‐derived cues into context‐dependent cellular responses. Hippo signaling is responsive to diverse upstream stimuli—including GPCR ligands, mechanical and cytoskeletal cues, growth factors, and metabolic stress—many of which originate from systemic sources such as circulating hormones, nutrients, and microbial metabolites [24, 25, 26, 27, 28, 29]. This systemic responsiveness positions Hippo‐YAP/TAZ as a plausible interface between systemic conditions and tissue‐specific transcriptional outputs. In this review, we explore the emerging concept of Hippo signaling as a systemic communication network, focusing on the gut, adipose tissue, and skeletal muscle as representative axes.

## Bidirectional communication between gut and liver

The gut–liver axis forms a bidirectional communication network that enables the liver to sense and respond to intestinal signals [30, 31, 32]. The liver receives approximately 70% of its blood supply from the intestine through the portal vein, making it the first organ exposed to gut‐derived metabolites, microbial products, and hormones [33, 34, 35, 36]. This unique connection positions the liver as a central hub for integrating systemic signals. Among these, fibroblast growth factor 15/19 (FGF15/19) and the gut microbiota have emerged as two major upstream regulators of hepatic Hippo signaling, linking intestinal physiology to liver homeostasis and disease.

### FGF15/19: An endocrine activator of hepatic Hippo signaling

The fibroblast growth factor (FGF) family is classified into three functional types: intracrine, paracrine, and endocrine [37]. Among them, FGF15 in mice and its human ortholog FGF19 belong to the endocrine subgroup. Unlike most FGFs, which act locally near their site of production through high‐affinity binding to heparin/heparan sulfate proteoglycans (HSPGs), FGF15/19 has low binding affinity for heparin/heparan sulfate [38]. This property allows them to escape from the extracellular matrix and circulate systemically, functioning as endocrine hormones that act on distant organs.

**Fig. 2:**
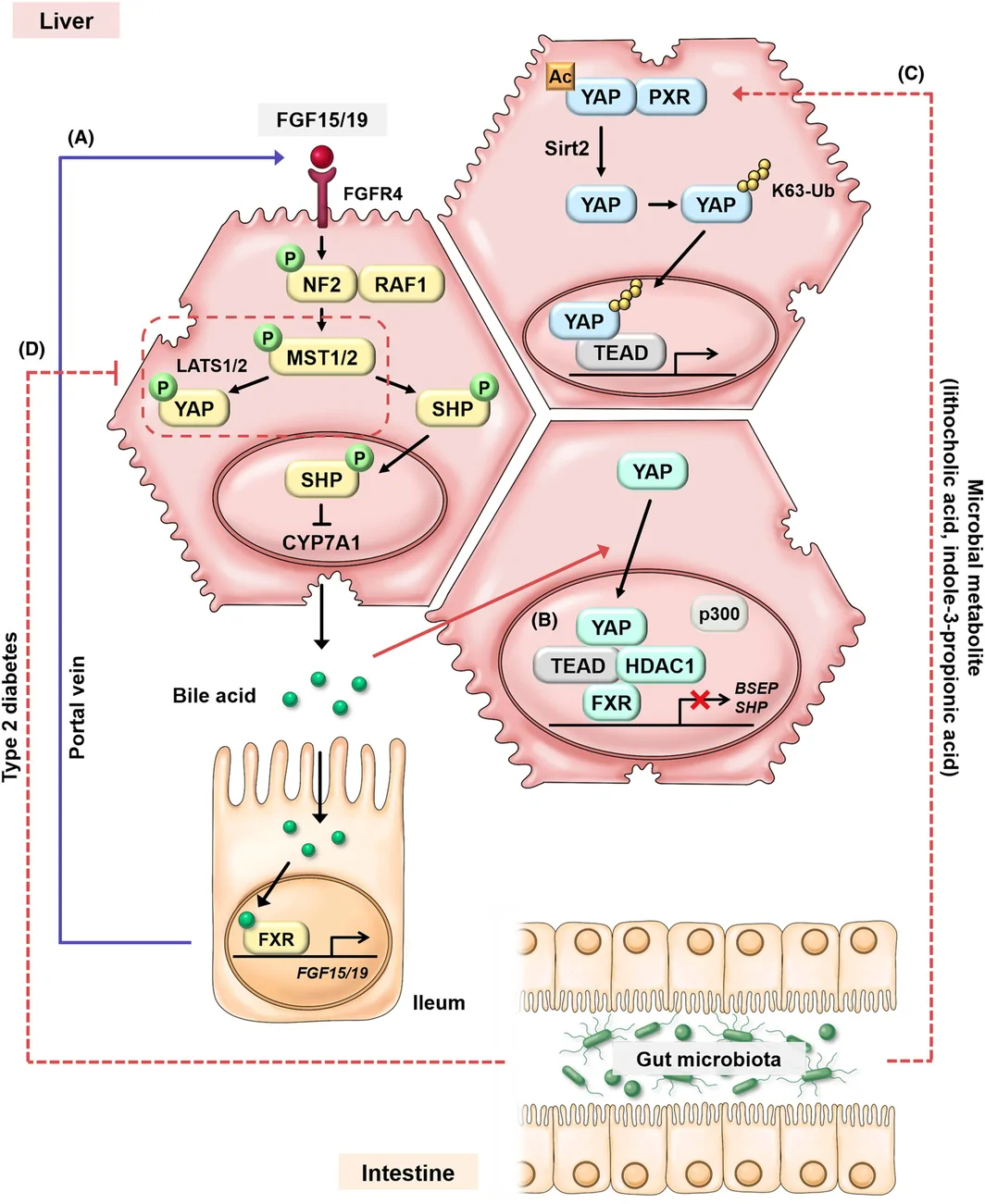
Gut‐derived FGF15/19 and microbiota signals regulate hepatic Hippo signaling. This schematic diagram illustrates how endocrine and microbial cues originating from the gut modulate the hepatic Hippo‐YAP pathway. (A) Following food intake, bile acids are secreted into the intestine and absorbed in the ileum. In enterocytes, bile acids activate the nuclear receptor FXR, inducing the secretion of FGF15/19 into the portal circulation. In hepatocytes, FGF15/19 binds to FGFR4, activating the canonical MST1/2‐LATS1/2 kinase cascade. This cascade phosphorylates YAP, preventing its nuclear localization and transcriptional activity. In parallel, activated MST1/2 phosphorylates and stabilizes SHP, which represses CYP7A1 expression and thereby suppresses bile acid synthesis. (B) In hepatocytes, YAP inhibits the FXR‐dependent transcriptional program. YAP is recruited to FXR by TEAD transcription factors and decreases SHP and BSEP expression by promoting the replacement of the transcription coactivator p300 with the histone deacetylase HDAC1. Altered bile acid signals further increase hepatic YAP activity. (C) Under xenobiotic stress, PXR agonists such as PCN or rifampicin can alter gut microbial composition. The resulting microbial metabolites amplify hepatic PXR‐YAP signaling. Activated PXR directly interacts with YAP, promoting Sirt2‐mediated deacetylation. Deacetylated YAP undergoes K63‐linked ubiquitination, translocates into the nucleus, and binds to TEAD transcription factors to induce the expression of target genes. (D) In type 2 diabetes, chronic dysbiosis suppresses the canonical Hippo kinase pathway in the liver. This suppression is characterized by reduced protein levels of the core kinases MST1/2 and LATS1/2. The loss of these upstream kinases results in a lower phospho‐YAP/YAP ratio, thereby stabilizing YAP. Solid lines represent established mechanisms, while dashed lines indicate proposed or incompletely defined mechanisms. Blue lines denote signals that activate Hippo signaling and inhibit YAP activity, while red lines indicate signals that suppress Hippo signaling and activate YAP.

Secretion of FGF15/19 is tightly coupled to the enterohepatic circulation of bile acids [39]. Bile acids, synthesized from cholesterol in the liver and stored in the gallbladder, are secreted into the duodenum after feeding to facilitate lipid absorption. Following digestion, bile acids are reabsorbed in the ileum, where they activate the nuclear receptor farnesoid X receptor (FXR), which in turn induces FGF15/19 expression [40, 41, 42]. The secreted FGF15/19 moves through the portal vein to the liver and binds to the FGFR4/β‐Klotho complex on hepatocytes [43, 44], suppressing CYP7A1—the rate‐limiting enzyme for bile acid synthesis—and thereby maintaining bile acid homeostasis [45] (Fig. 2A).

A recent study revealed that this classical bile acid feedback loop is, at least in part, mediated by the Hippo pathway. Upon FGF15–FGFR4 activation, NF2 is phosphorylated and binds RAF1, an inhibitor of MST1/2. This sequestration releases MST1/2 from RAF1‐mediated suppression, thereby activating the MST1/2‐LATS1/2 kinase cascade. Activated MST1/2 indirectly induces YAP phosphorylation via LATS1/2, while directly phosphorylating the small heterodimer partner (SHP). Phosphorylation of SHP prevents its ubiquitination and degradation, thereby stabilizing it. Stabilized SHP inhibits CYP7A1 transcription in the nucleus (Fig. 2A, Table 1). This mechanism identifies Hippo signaling as a central effector of FGF15‐mediated bile acid homeostasis [46].

Dysregulation of this feedback loop disrupts hepatic sensing of intestinal cues, leading to uncontrolled bile acid synthesis and accumulation of hepatotoxic bile acids. This chronic overload provokes hepatocellular injury, inflammation, and fibrosis, driving the development of cholestatic liver disease [39, 45, 47] and non‐alcoholic steatohepatitis [48].

In addition to this endocrine feedback mechanism, bile acid homeostasis is also regulated at the hepatocyte level. Whereas ileal FXR inhibits bile acid synthesis indirectly by inducing FGF15/19, hepatic FXR directly regulates bile acid synthesis and export through transcriptional activation of target genes, including SHP and bile salt export pump (BSEP) [40, 49]. Liu *et al*. further showed that YAP suppresses this hepatic FXR‐dependent transcriptional program, thereby disrupting bile acid homeostasis. Mechanistically, TEAD transcription factors bind FXR and recruit YAP, promoting a cofactor switch from transcription coactivator p300 to the histone deacetylase HDAC1 [50] (Fig. 2B). Together, these findings extend the classical intestinal FXR‐FGF15/19 feedback loop by adding a hepatocyte‐intrinsic YAP‐FXR regulatory circuit.

Beyond its homeostatic role, FGF15 also functions as a mitogenic and regenerative signal. In Fgf15 transgenic mice, hepatocytes exhibited increased Cyclin D1 and PCNA expression and accelerated proliferation following partial hepatectomy. This enhanced proliferation is primarily mediated by STAT3 activation. In parallel, NF‐κB signaling induces immediate‐early genes (*c‐Jun*, *c‐Fos*) that prime hepatocytes for cell‐cycle entry and promote cell survival. In addition, MAPK pathway activation contributes to the suppression of bile acid synthesis, a crucial step that prevents bile acid toxicity and protects the regenerating liver from cholestatic injury. Interestingly, regeneration terminated earlier in these mice, accompanied by increased YAP phosphorylation. Thus, FGF15 functions both as an accelerator—promoting regeneration through STAT3, NF‐κB, and MAPK pathways—and as a brake—terminating growth through YAP inhibition [51].

Collectively, these studies identify FGF15/19 as an endocrine activator of hepatic Hippo signaling that preserves homeostasis, prevents excessive growth, and restrains tumorigenesis.

### Gut microbiota: A microbial modulator of hepatic H ippo signaling

Beyond hormonal regulation, the gut microbiota provides diverse microbial and metabolic cues that influence hepatic Hippo signaling. The microbiota produces metabolites, bile acid derivatives, and microbial‐associated molecular patterns (MAMPs) that reach the liver via the portal circulation [31, 34, 52, 53]. Dysregulation of these gut‐derived signals critically contributes to chronic liver diseases, including non‐alcoholic fatty liver disease (NAFLD), NASH, and hepatocellular carcinoma (HCC) [54, 55, 56]. Although direct mechanistic evidence remains limited, emerging studies in distinct pathological contexts—such as xenobiotic responses and type 2 diabetes—suggest that microbiota‐derived signals can modulate hepatic Hippo signaling [57, 58].

Gut microbiota amplifies the hepatic responses to xenobiotic stress through the pregnane X receptor (PXR)–YAP axis (Fig. 2C). PXR is a nuclear receptor that senses xenobiotics and regulates the transcription of enzymes involved in drug metabolism and detoxification [59]. PXR agonists, such as pregnenolone 16α‐carbonitrile (PCN) or rifampicin (RIF), promote an interaction between PXR and YAP, mediated by the ligand‐binding domain (LBD) of PXR and the WW domain of YAP. This interaction activates YAP through a non‐canonical mechanism that operates independently of the MST1/2–LATS1/2 cascade. Upon activation, PXR recruits the deacetylase Sirt2, which removes p300‐mediated acetylation of YAP and switches its ubiquitination pattern from K48‐linked (proteolytic) to K63‐linked (non‐proteolytic) ubiquitination. Although the subcellular site of YAP acetylation in this context remains undefined, Sirt2‐dependent deacetylation facilitates YAP stabilization and nuclear translocation. In the nucleus, YAP binds to the TEAD transcription factor to induce proliferative gene programs. Collectively, this cascade drives hepatocyte enlargement and proliferation, thereby contributing to hepatomegaly [60, 61].

Importantly, this PXR–YAP activation requires an intact gut microbiota. In antibiotic‐treated (ABX) mice, PCN‐induced hepatomegaly and YAP activation were markedly attenuated, whereas fecal microbiota transplantation (FMT) from PCN‐treated donors restored both phenotypes. Mechanistically, microbiota depletion significantly reduced the expression of PXR target genes (CYP3A11, CYP2B10) and YAP target genes (CTGF, CYR61, ANKRD1), while retaining YAP in an inactive, cytoplasmic state. These findings indicate that gut microbiomes are essential for activation of the hepatic PXR–YAP axis [57].

Although the specific microbial metabolites remain undefined, PCN‐induced alterations in microbiota composition may influence the availability of microbiota‐derived PXR ligands, potentially enhancing hepatic PXR signaling and downstream YAP activation. Candidate metabolites include lithocholic acid and indole‐3‐propionic acid, both of which have been reported as PXR ligands [62, 63]. Together, these xenobiotic and microbiota‐derived signals synergistically amplify hepatic PXR–YAP activation, establishing a mechanistic link between microbial metabolism, xenobiotic stress, and liver enlargement.

Further evidence from db/db mice, a model of type 2 diabetes, demonstrated systemic suppression of Hippo signaling under chronic metabolic stress. db/db mice exhibited reduced MST1 and LATS1 protein levels and lower phospho‐YAP/YAP ratios in the liver [58]. Fecal transplantation from db/db mice donors recapitulated both metabolic phenotypes and Hippo suppression in recipient mice. Notably, these changes extended beyond the liver to the heart, kidney, and muscle, indicating that microbial signals can exert widespread systemic effects. Although the exact mediators remain unknown, these findings suggest that microbiota from db/db mice are associated with reduced expression of upstream Hippo kinases and enhanced YAP activity across multiple organs [58] (Fig. 2D).

Together, these findings emphasize that the Hippo–YAP pathway serves as a key integrator of endocrine and microbial signals within the gut–liver axis. While FGF15/19 activates Hippo signaling to suppress YAP and maintain hepatic homeostasis, microbial cues inhibit Hippo signaling, leading to YAP activation and pathological outcomes. Thus, Hippo signaling functions as a molecular switch that interprets and translates gut‐derived physiological and pathological cues into appropriate hepatic responses.

## Pathogenic communication between gut and pancreas

The pancreas, composed of both endocrine and exocrine compartments, plays essential roles in digestion and glucose regulation [64]. Due to its close anatomical and physiological proximity to the intestine, it is highly susceptible to gut‐derived metabolites, bile acids, and microbial products that reach it through the bloodstream or biliary system [65, 66].

Gut dysbiosis disrupts this communication through multiple mechanisms. First, a damaged intestinal barrier allows the translocation of microbial products such as lipopolysaccharide (LPS) into the circulation, triggering systemic inflammation [67, 68]. Second, dysbiosis alters key microbial enzymatic activities required for bile acid metabolism—such as bile acid deconjugation and 7α‐dihydroxylation—thereby disturbing the composition of the bile acid pool [69, 70]. These alterations generate a pro‐inflammatory and tumor‐promoting environment that can affect distal organs, including the pancreas [66, 68, 71].

This vulnerability is evident across distinct disease contexts. In acute pancreatitis, increased translocation of microbial products aggravates systemic inflammation and pancreatic injury [72, 73, 74, 75]. In type 1 diabetes, dysbiosis alters immune tolerance and triggers autoimmune responses against pancreatic β‐cells [76, 77, 78]. Mechanistically, impaired intestinal barrier integrity allows microbial products such as LPS to enter the circulation, leading to continuous low‐grade systemic inflammation—a state of persistent immune activation that keeps inflammatory signaling switched on [79]. At the same time, dysbiosis reduces the production of short‐chain fatty acids (SCFAs), key microbial metabolites that normally promote the differentiation and maintenance of regulatory T cells (Tregs), which act as suppressors of excessive immune activation [80]. The loss of these SCFA‐derived anti‐inflammatory signals weakens Treg‐mediated immune restraint. Consequently, an immune system that is chronically activated but lacks adequate regulation becomes prone to attacking self‐antigens, ultimately initiating the autoimmune destruction of pancreatic β‐cells characteristic of type 1 diabetes [81].

While these examples highlight the physiological importance of gut–pancreas communication and its dysregulation in disease, the direct mechanistic involvement of Hippo signaling within this axis has so far been demonstrated only in pancreatic ductal adenocarcinoma (PDAC) [82]. Nearly all PDAC cases are driven by oncogenic KRAS [83], and YAP functions as a synergistic co‐driver rather than a parallel effector. KRAS‐dependent activation of MAPK, PI3K, and Rho GTPase signaling enhances YAP activity, which in turn amplifies metabolic and survival programs via YAP–TEAD transcriptional output [84, 85, 86, 87].

**Fig. 3:**
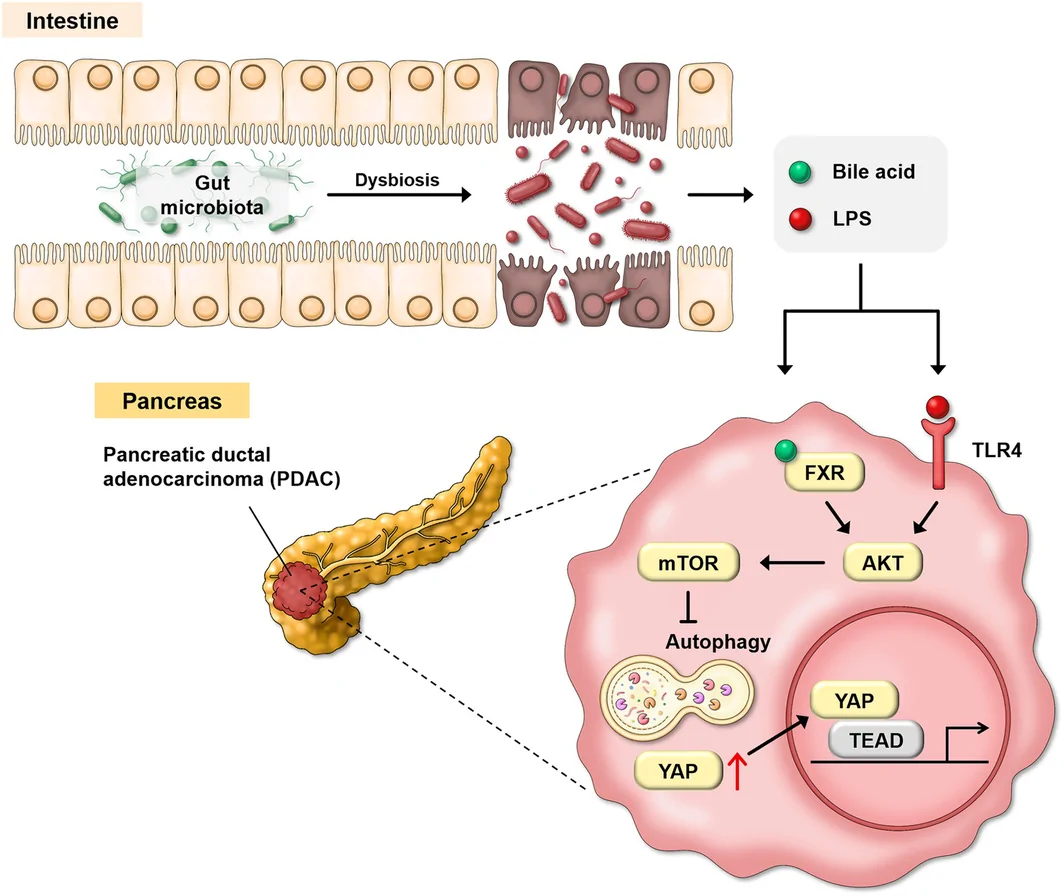
Gut‐derived inflammatory and bile acid signals activate YAP through a non‐canonical mechanism in the pancreas. This schematic shows how gut‐derived inflammatory and metabolic cues activate YAP in pancreatic cells via a non‐canonical, autophagy‐dependent pathway. Gut dysbiosis impairs intestinal barrier integrity, allowing microbial products such as lipopolysaccharide (LPS) to enter the bloodstream and alters key microbial enzymatic activities necessary for bile acid metabolism, disrupting the composition of the bile acid pool. In pancreatic ductal adenocarcinoma (PDAC), LPS binds to toll‐like receptor 4 (TLR4), and bile acids activate the farnesoid X receptor (FXR). Both pathways converge on the AKT–mTOR pathway, leading to inhibition of autophagy. Blocking autophagic flux prevents YAP degradation, stabilizes it, promotes its nuclear translocation, and activates transcription.

A recent study revealed that gut‐derived inflammatory and bile acid signals modulate Hippo signaling through non‐canonical, autophagy‐dependent mechanisms in PDAC (Fig. 3, Table 1). Specifically, LPS activates toll‐like receptor 4 (TLR4), and bile acids activate FXR, both converging on the AKT–mTOR pathway to inhibit autophagic flux. Autophagy suppression prevents YAP degradation and enables its nuclear translocation, leading to the activation of transcriptional programs related to proliferation and fibrosis. These effects occur within the inflammation‐driven context established by LPS and bile acids, thereby creating a tumor‐promoting environment [82].

Supporting this mechanism, *in vivo* experiments showed that systemic LPS administration or bile duct ligation accelerates pancreatic intraepithelial neoplasia (PanIN) progression in KRAS‐driven PDAC mouse models [82]. Both interventions impair autophagic flux, leading to YAP accumulation. Conversely, treatment with cholestyramine—a bile acid and endotoxin sequestrant—reduced both LPS and bile acid levels. This intervention restored autophagic flux, enhanced YAP degradation, and consequently suppressed tumor growth. [82]. From this perspective, gut‐derived LPS and bile acid signaling act as amplifiers of the KRAS oncogenic network by stabilizing YAP, thereby linking microbial dysbiosis to enhanced tumor progression.

Collectively, these findings reveal a dual regulatory pattern between organ systems. In the liver, endocrine cues such as FGF15/19 activate the canonical MST–LATS cascade to suppress YAP and maintain homeostasis, whereas in the pancreas, gut‐derived inflammatory and bile acid signals inhibit autophagy to stabilize YAP and promote tumorigenesis. This contrast emphasizes Hippo signaling as a dynamic integrator of organ‐specific inputs, translating physiological or pathological cues into distinct outcomes—homeostatic suppression in the liver versus pro‐tumorigenic activation in the pancreas.

Despite these insights, several key questions remain unresolved. Within the PDAC, the specific microbial products and bile acid species that most strongly influence YAP stability are yet to be defined. The precise causal relationships between this gut‐induced YAP stabilization and KRAS‐driven transcriptional programs—particularly those governing fibrosis and immune evasion—also require clarification. More broadly, whether this non‐canonical, autophagy‐dependent YAP regulation represents a shared pathological mechanism across other gut–pancreas axis disorders remains unknown. Addressing these gaps will be crucial for understanding how gut‐derived signals reshape pancreatic physiology and pathology through the Hippo–YAP pathway, potentially revealing new therapeutic targets across a spectrum of pancreatic diseases.

## Endocrine communication between adipose tissue and distant organs

Adipose tissue, traditionally viewed as a passive energy storage, is now recognized as a dynamic endocrine organ that communicates systemic metabolic states through the secretion of adipokines [88, 89, 90]. These adipokines act as endocrine messengers that transmit information about the nutritional and inflammatory status of adipose tissue to distant organs, including the brain, liver, and skeletal muscle [88, 90, 91, 92]. Under chronic caloric excess, however, adipose tissue becomes dysfunctional—a condition referred to as adiposopathy or sick fat [93]. This dysfunction is characterized by adipocyte hypertrophy, visceral fat accumulation, and an altered secretory profile enriched in free fatty acids and pro‐inflammatory adipokines [94]. Such changes are key contributors to obesity‐related diseases, such as metabolic syndrome, type 2 diabetes, and cancers [6, 95, 96]. Within this systemic context, Hippo signaling connects local adipocyte regulation with systemic endocrine communication, acting both as a generator and a responder of adipokine‐mediated signals.

### Leptin and Vaspin: Opposing systemic modulators of Hippo signaling

**Fig. 4:**
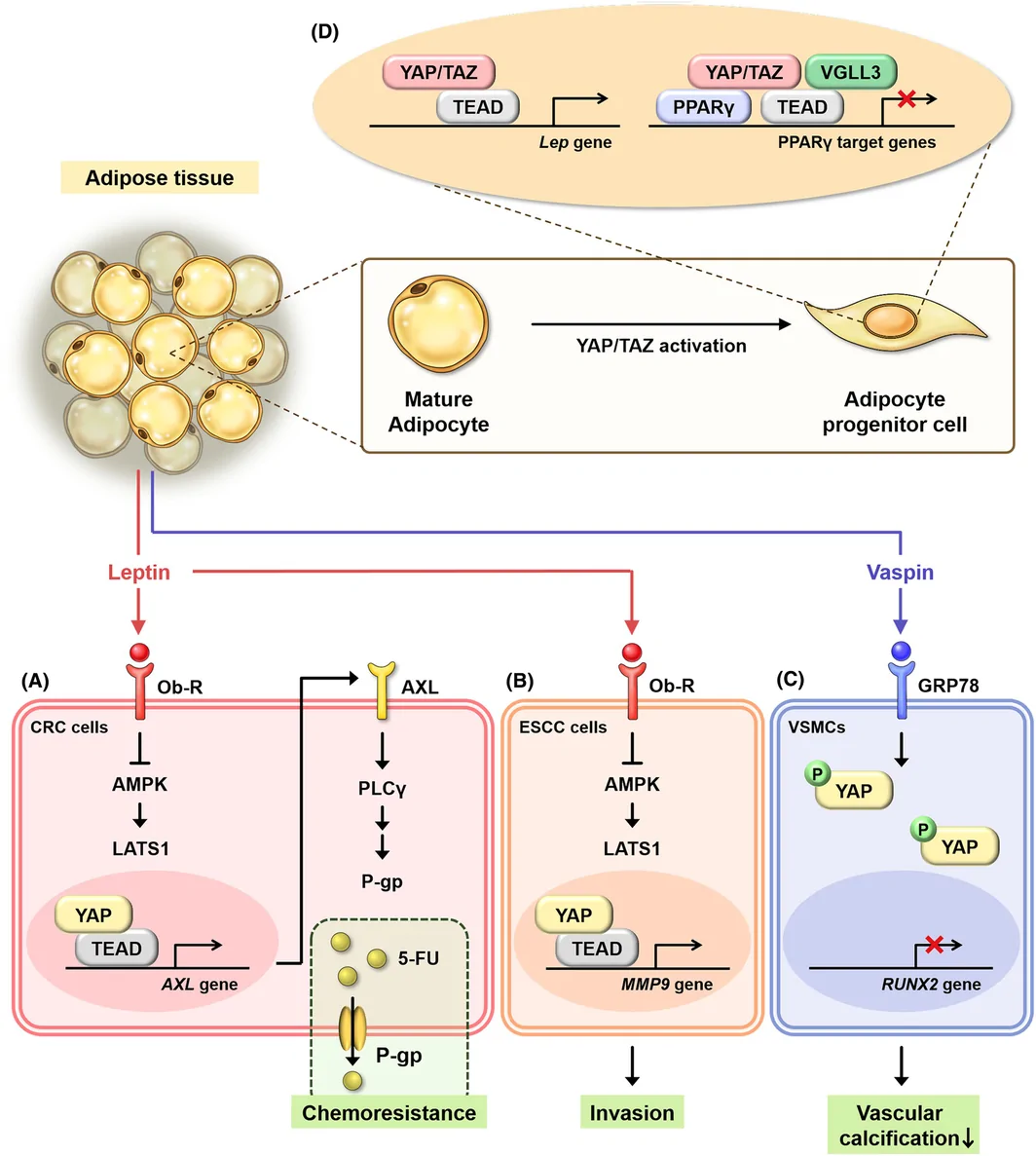
Hippo signaling links local adipocyte regulation with systemic endocrine signaling. This schematic diagram shows how hippo signaling functions both as a generator and as a target of adipokine‐mediated endocrine signaling. (A, B) Leptin, secreted from adipose tissue, suppresses the AMPK‐LATS pathway in colorectal cancer and esophageal squamous cell carcinoma (ESCC) cells, leading to YAP activation. (A) In colorectal cancer cells, nuclear YAP induces AXL expression, which activates PLCγ signaling and increases P‐glycoprotein (P‐gp), an ATP‐dependent drug efflux pump for 5‐fluorouracil (5‐FU), resulting in chemoresistance. (B) In ESCC cells, YAP activation induces MMP9 expression, promoting tumor invasion. (C) Vaspin—a serine protease inhibitor secreted from visceral adipose tissue—binds to the GRP78 receptor in vascular smooth muscle cells (VSMCs), thereby enhancing YAP phosphorylation and retention in the cytoplasm. This YAP inactivation prevents osteogenic gene expression and offers protection against vascular calcification. (D) In adipocytes, nuclear YAP/TAZ suppresses the PPARγ adipogenic program in a TEAD‐dependent way. VGLL3 functions as a downstream effector that strengthens this suppression, promoting adipocyte dedifferentiation. In parallel, the YAP/TAZ‐TEAD complex directly binds the *Lep* enhancer to promote leptin transcription.

Among adipokines, leptin and vaspin exert opposing systemic effects on Hippo signaling—one promoting cancer and the other providing protection (Fig. 4).

Leptin, one of the most well‐characterized adipokines, exemplifies how adipose‐derived signals can regulate Hippo signaling in distant organs. Elevated leptin levels in obesity act as systemic cues that modulate YAP activity and promote tumor progression. Conditioned media from obese adipocytes, enriched in leptin, induced resistance to 5‐fluorouracil (5‐FU) in colorectal cancer (CRC) cells. This effect was abolished by leptin‐neutralizing antibodies and recapitulated by recombinant leptin treatment, confirming leptin as a direct mediator [97]. Mechanistically, leptin inhibits AMPK activity, thereby reducing LATS1 phosphorylation, leading to YAP dephosphorylation and nuclear translocation. Nuclear YAP forms a complex with TEAD to induce the expression of AXL, a receptor tyrosine kinase that promotes cancer cell survival and drug resistance [98, 99] (Fig. 4A, Table 1). Activated AXL recruits and activates phospholipase C gamma (PLCγ), initiating a downstream signaling cascade that enhances the expression of P‐glycoprotein (P‐gp). P‐gp, an ATP‐dependent efflux pump belonging to the ATP‐binding cassette (ABC) transporter family, actively exports a wide range of chemotherapeutic agents from the cytoplasm. As a result, elevated P‐gp levels reduce the intracellular accumulation of drugs such as 5‐FU, a known P‐gp substrate, thereby reducing their cytotoxic efficacy and conferring chemoresistance [100, 101] (Fig. 4A).

A similar mechanism was reported in esophageal squamous cell carcinoma (ESCC), where diet‐induced obesity promotes tumor growth and invasion. This phenotype was accompanied by decreased AMPK activity, YAP activation, and increased expression of the invasion‐related gene MMP9 [102] (Fig. 4B, Table 1). Collectively, these studies suggest that obesity‐related hyperleptinemia activates YAP, linking metabolic imbalance to tumor progression. Notably, most of these findings were derived from subcutaneous xenograft models or CRC and ESCC, which do not fully recapitulate the native tumor microenvironment. Validation in orthotopic or genetically engineered tumor models under obesity‐associated conditions will be essential to confirm the physiological relevance of leptin–YAP signaling *in vivo*. Although direct evidence for the leptin‐YAP axis is currently limited to colorectal and esophageal cancers, it may represent a broader mechanism underlying obesity‐associated malignancies. Given the well‐established role of leptin as a systemic link between obesity and tumor progression [103, 104, 105], similar YAP‐dependent processes are likely to operate in other contexts, including breast, pancreatic, and liver cancers.

In contrast, vaspin is a serine protease inhibitor secreted from visceral adipose tissue. Notably, its expression is often upregulated in obesity and insulin resistance, likely as a compensatory response to the metabolic dysfunction [106, 107, 108]. Vaspin exerts protective effects that appear to be mediated, at least in part, through the Hippo signaling pathway [109].

The long non‐coding RNA LEF1‐AS1, which is upregulated in patients with atherosclerosis, suppresses YAP phosphorylation through an undefined mechanism. As a result, dephosphorylated YAP translocates into the nucleus, where it induces osteogenic genes such as RUNX2, driving vascular smooth muscle cell (VSMC) differentiation and calcium deposition [109]. Conversely, vaspin counteracts this process by binding to the glucose‐regulated protein 78 (GRP78) receptor [110], enhancing YAP phosphorylation and thereby retaining YAP in the cytoplasm to prevent osteogenic transcription. Consequently, RUNX2 expression and vascular calcification were reduced [109] (Fig. 4C).

Vascular calcification—a pathological process closely associated with atherosclerosis and cardiovascular mortality [111, 112]—is aggravated by metabolic dysfunction and chronic inflammation, both hallmark features of insulin‐resistant states [113]. Notably, vaspin secretion increases under these conditions, likely as a compensatory mechanism that protects the vasculature [106, 107]. In this context, vaspin inhibits YAP to suppress pathological osteogenic differentiation, thereby counteracting vascular calcification. This protective function may extend beyond calcification to contribute to broader cardiovascular homeostasis.

Taken together, leptin and vaspin indicate the duality of adipose‐derived regulation of YAP activity—leptin promotes tumor progression by activating YAP, whereas vaspin preserves vascular health through its inhibition.

### Hippo signaling control of adipose fate and leptin regulation

While leptin and vaspin demonstrate the systemic consequences of the adipokine–Hippo axis, the Hippo pathway within adipocytes determines how these endocrine signals are generated.

Within adipocytes, Hippo signaling regulates both the plasticity of mature adipocytes and adipokine expression (Fig. 4D). In adipocyte‐specific Lats1/2 knockout mice, constitutive activation of YAP/TAZ drove mature adipocytes to dedifferentiate into progenitor‐like cells, resulting in lipoatrophy [114]. This dedifferentiation resulted from YAP/TAZ‐mediated suppression of peroxisome proliferator‐activated receptor‐γ (PPARγ) target genes, which are essential for maintaining adipocyte identity [115, 116, 117, 118]. Pharmacological activation of PPARγ with rosiglitazone [119] restored adipocyte differentiation, indicating the intrinsic plasticity and reversibility of adipocyte fate [114].

Mechanistically, recent work has shown that YAP/TAZ suppress adipocyte differentiation not primarily through direct binding to and co‐repression of PPARγ, but through TEAD‐dependent chromatin repression of the *Pparg2* locus and adipogenic enhancers. In this model, YAP/TAZ minimally affected PPARγ genomic occupancy but markedly reduced chromatin accessibility and H3K27ac at PPARγ‐bound adipogenic enhancers, while VGLL3 acted as a critical downstream effector that reinforces transcriptional repression through HDAC3 co‐repressor complexes [120].

In addition to regulating adipocyte fate, YAP/TAZ also regulate leptin expression. Despite a significant reduction in fat mass, adipocyte‐specific Lats1/2 knockout mice displayed paradoxically elevated circulating leptin levels [114]. This observation challenges the conventional notion that circulating leptin levels directly correlate with adipose mass [121]. Mechanistically, the YAP/TAZ–TEAD complex binds a conserved enhancer region upstream of the *Lep* gene, directly promoting leptin transcription.

Importantly, the role of YAP/TAZ extends beyond this genetic model to physiological contexts. In wild‐type mice, refeeding after fasting activated YAP/TAZ signaling in adipose tissue, as evidenced by a marked decrease in the phospho‐YAP/YAP ratio and increased nuclear localization of YAP/TAZ in adipocytes. Functionally, adipose‐specific YAP/TAZ knockout mice failed to induce *Lep* expression upon refeeding, demonstrating that this pathway is required for the adaptive regulation of leptin in response to nutritional changes. These findings establish Hippo‐YAP/TAZ signaling as a physiological mediator that translates systemic energy status into hormonal outputs from adipose tissue [114].

Functionally, YAP/TAZ activation in adipocytes exerts systemic metabolic effects. In Lats1/2‐deficient adipocytes, constitutive YAP/TAZ activity maintained high circulating leptin levels despite severe lipoatrophy, which was essential for preventing the metabolic dysfunction. Elevated leptin increased systemic energy expenditure and enhanced hepatic fatty acid oxidation, which in turn prevented the ectopic lipid accumulation that is the primary cause of insulin resistance in lipodystrophy, thereby preserving metabolic homeostasis despite their lack of adipose depots [114].

Thus, Hippo signaling within adipocytes coordinates adipose tissue mass and systemic metabolism through two independent pathways: (a) the YAP/TAZ‐PPARγ axis, which regulates adipocyte identity and plasticity to determine fat storage capacity; and (b) the YAP/TAZ‐TEAD axis, which promotes leptin transcription to control systemic energy expenditure. Together, these mechanisms position the Hippo pathway as both a local determinant of adipocyte fate and a molecular bridge linking adipose tissue with systemic metabolic regulation.

## Metabolic communication between muscle, adipose tissue, and liver

Skeletal muscle, which accounts for nearly 40% of total body mass, serves as a central regulator of systemic energy balance through glucose uptake and fatty acid oxidation [122, 123, 124]. Beyond this metabolic role, skeletal muscle also functions as an endocrine organ that communicates with other metabolic organs—particularly adipose tissue and the liver—through secreted myokines and metabolites [122, 125, 126]. These interorgan interactions form a muscle–adipose–liver axis that coordinates whole‐body energy utilization and storage. Disruption of this communication in skeletal muscle impairs systemic metabolic balance and contributes to the development of metabolic disorders such as type 2 diabetes [127, 128, 129]. Within this interorgan network, a recent study identified the Hippo pathway effector YAP as a critical factor, demonstrating that its local function in maintaining muscle metabolic efficiency is essential for systemic energy homeostasis.

**Fig. 5:**
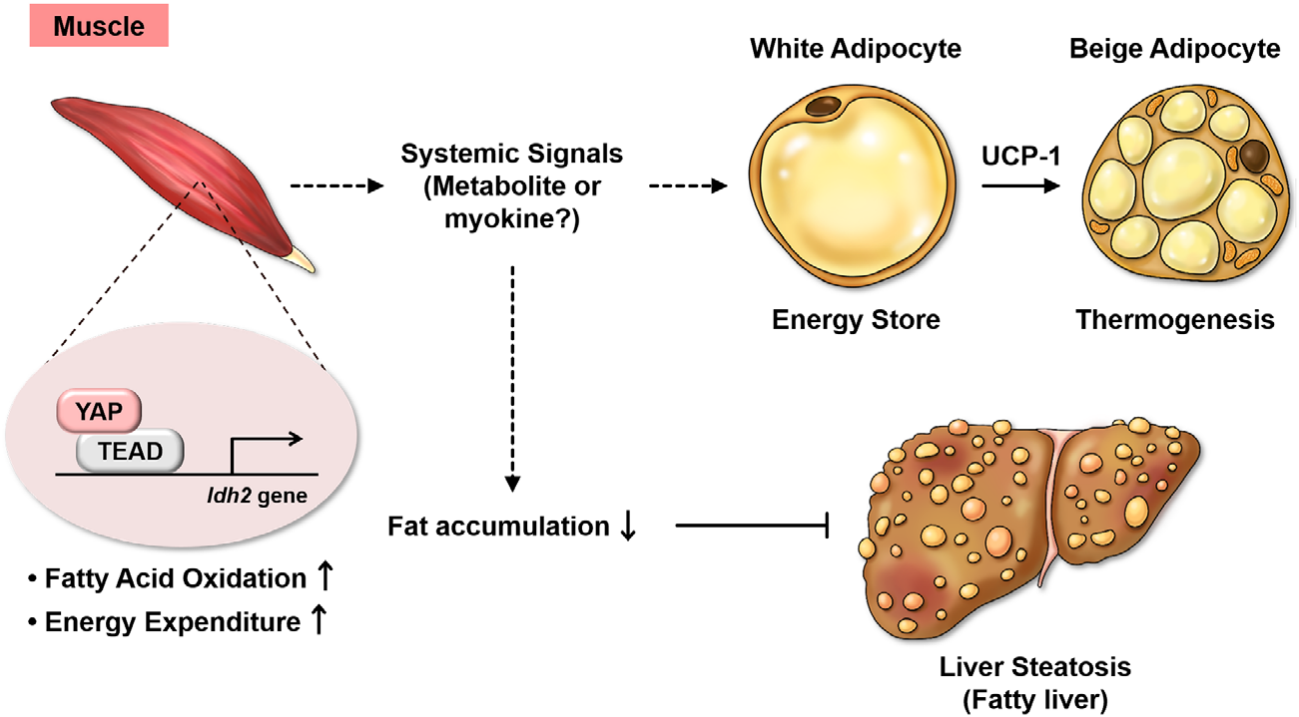
Outcomes of YAP signaling derived from skeletal muscle contribute to systemic regulation. This schematic illustrates how organ‐specific Hippo–YAP activity in skeletal muscle causes systemic effects. In skeletal muscle, YAP activation induces *Idh2* expression, increasing tricarboxylic acid (TCA) cycle flux, fatty acid oxidation, and energy expenditure. YAP‐driven muscle activity is also thought to stimulate the release of an unidentified myokine, which may act on peripheral metabolic organs to promote white adipose tissue (WAT) browning and reduce hepatic lipid accumulation, thereby protecting against hepatic steatosis (fatty liver). Solid lines represent established mechanisms, while dashed lines indicate proposed or incompletely defined mechanisms.

In obese and insulin‐resistant patients and corresponding mouse models, reduced YAP protein levels were observed in skeletal muscle [130]. Mechanistically, muscle‐specific deletion of YAP reduces the expression of genes involved in the tricarboxylic acid (TCA) cycle, including isocitrate dehydrogenase 2 (IDH2) (Fig. 5). IDH2 is a key mitochondrial enzyme that catalyzes the oxidative decarboxylation of isocitrate to α‐ketoglutarate within the TCA cycle. Its loss leads to incomplete fatty acid oxidation—a pathological mismatch between β‐oxidation rates and the capacity of the TCA cycle, resulting in lipid accumulation and lipotoxic stress [130]. These findings suggest that YAP is an essential regulator of efficient substrate utilization within skeletal muscle. Conversely, restoring YAP expression specifically in the skeletal muscle of obese db/db mice improved systemic metabolism. This intervention reduced fat mass, increased overall energy expenditure, and protected against hepatic steatosis, all without changing lean mass [130].

Notably, muscle‐specific YAP activation induced the emergence of beige adipocytes within white adipose tissue (WAT)—the body's primary site for energy storage [130]. Unlike white adipocytes that contain a single large lipid droplet, beige adipocytes possess multiple small lipid droplets and express uncoupling protein 1 (UCP1) [131]. This enables them to dissipate energy as heat through thermogenesis rather than store it. This remodeling of WAT toward an energy‐expending phenotype highlights the ability of muscle‐derived YAP activity to exert endocrine‐like effects on adipose tissue. In parallel, improved muscle metabolism likely alleviated systemic lipid overload, indirectly preventing hepatic fat accumulation and maintaining liver homeostasis [130]. This protection against hepatic steatosis indicates a functional muscle–liver axis. Collectively, these findings identify skeletal muscle YAP as a key modulator of systemic energy balance through interorgan communication with adipose tissue and the liver (Fig. 5). Although the direct mediators downstream of skeletal muscle YAP remain undefined, muscle‐derived circulating factors such as β‐aminoisobutyric acid (BAIBA), irisin, and meteorin‐like (Metrnl) can be considered as plausible candidates that could explain the observed adipose browning and protection against hepatic steatosis (Table 2). However, none of these factors has been shown to be regulated by YAP, and this possibility requires further investigation.

**Table 2:** Selected candidates potentially related to the unidentified mediator downstream of skeletal muscle YAP.

Candidate	Classification	Relevance to adipose browning	Relevance to protection against hepatic steatosis	Reference
β‐aminoisobutyric acid (BAIBA)	Muscle‐derived metabolite	Induces the expression of brown adipocyte‐specific genes in white adipocytes	Induces hepatic β‐oxidation gene expression; direct anti‐steatotic effects have been reported in some mouse models	[148, 149, 150]
Irisin	Myokine	Promotes white adipocyte browning	Suppresses hepatocellular lipogenesis and lipid accumulation in a PRMT3‐dependent manner	[151, 152]
Meteorin‐like (Metrnl)	Adipomyokine	Stimulates beige fat thermogenesis in white adipose tissue	Alleviates hepatic steatosis in NAFLD mouse models by regulating hepatic triglyceride secretion and fatty acid oxidation	[153, 154]

Taken together, these results extend the concept introduced in adipose tissue, demonstrating that Hippo signaling can function not only as a recipient of systemic cues but also as a generator of systemic outputs. By coupling local metabolic control with long‐range physiological regulation, muscle Hippo–YAP signaling emerges as a central coordinator of the muscle–adipose–liver axis. Future studies aimed at identifying the YAP‐dependent myokines or metabolites that mediate these effects will be crucial for understanding how Hippo signaling contributes to systemic metabolic adaptation and for uncovering new therapeutic targets in obesity and metabolic disease.

## Therapeutic implications of Hippo signaling

As discussed in this review, dysregulated Hippo‐YAP/TAZ signaling is implicated in multiple disease contexts, including liver and metabolic disorders, chronic inflammatory and vascular diseases, and cancers. These findings suggest that Hippo signaling is not only a key mechanism of interorgan communication but also a potential therapeutic target in systemic disease. Because multiple systemic signals converge on YAP/TAZ, targeting YAP/TAZ may intercept a broader range of upstream inputs than inhibiting individual upstream regulators. Owing to the difficulty of directly targeting YAP/TAZ with small molecules [132], most pharmacological efforts have focused on disrupting YAP/TAZ‐TEAD interaction or inhibiting TEAD itself.

Verteporfin was an early pharmacological compound used to inhibit YAP‐driven transcription and has been used in preclinical studies, including hepatocellular carcinoma and PDAC [133, 134, 135]. However, the anti‐proliferative effects of verteporfin are not uniformly explained by direct and selective inhibition of the YAP‐TEAD complex [136, 137]. More selective next‐generation approaches have since been developed. Mechanistically, these agents represent distinct pharmacological strategies: IAG933 directly disrupts the YAP‐TEAD interface [138], whereas VT3989 inhibits TEAD palmitoylation, a modification important for TEAD stability and transcriptional activity [139, 140]. Notably, VT3989 has shown clinical antitumor activity in solid tumors, particularly mesothelioma [141].

In metabolic disease, however, Hippo‐YAP/TAZ‐directed therapy remains largely at the preclinical stage. Preclinical studies in MASLD/MASH suggest that suppressing YAP/TAZ activity may attenuate hepatic inflammation, as demonstrated by verteporfin treatment in diet‐induced NASH models [142], while hepatocyte‐targeted TAZ silencing has also shown antifibrotic effects in both mouse models and hepatocyte‐humanized liver systems [143, 144]. These studies support the YAP/TAZ‐TEAD axis as a therapeutic target and suggest that its relevance may extend beyond oncology.

## Conclusions

The Hippo signaling pathway has now emerged as a central hub that operates at the interface of systemic signaling and interorgan communication, extending its role beyond a master regulator of organ growth. Evidence from multiple organ axes indicates that Hippo signaling is not confined to cell‐intrinsic growth control but is engaged by organ‐derived signals, thereby influencing systemic physiology in both health and disease.

Systemic cues originating from the gut and adipose tissue demonstrate how Hippo signaling functions as a downstream interpreter of diverse physiological inputs. Gut‐derived mediators such as FGF15/19 and microbiota‐derived signals act as endocrine and microbial messengers that modulate Hippo signaling in distal organs through distinct mechanisms. In the liver, FGF15/19 activates the Hippo kinase cascade to suppress YAP and maintain bile acid homeostasis, whereas microbiota‐derived signals can activate YAP through metabolic stress and xenobiotic‐sensing‐related pathways. In the pancreas, inflammatory signaling via TLR4 and bile acid signaling via FXR converge on the AKT–mTOR pathway, leading to inhibition of autophagy and non‐canonical stabilization of YAP. Likewise, adipokines from adipose tissue can also regulate Hippo activity in distant target organs. Leptin enhances YAP activity to promote tumor progression, whereas vaspin suppresses YAP activation in vascular smooth muscle cells, thereby preserving tissue integrity. Together, these examples indicate how endocrine and microbial signals of systemic origin influence the Hippo–YAP pathway in a context‐dependent manner, positioning it as a responsive node within interorgan communication networks.

Conversely, Hippo signaling itself can generate systemic outputs that influence distant organs, highlighting its bidirectional involvement in interorgan communication. In adipose tissue, Hippo signaling regulates adipocyte fate and leptin expression, establishing an endocrine link that influences whole‐body energy balance. In skeletal muscle, YAP activity influences local metabolic programs and is linked to broader systemic metabolic effects, including protection against fatty liver disease and promotion of adipose tissue browning. These findings collectively suggest that Hippo signaling is not only a passive recipient of systemic inputs but also an active transmitter that propagates metabolic and endocrine information across organs.

Recent single‐cell and spatial transcriptomics studies further suggest that local cellular states and microenvironmental context shape the output of Hippo‐YAP/TAZ signaling. In the heart, YAP‐activated cardiomyocytes require a supportive fibroblast‐macrophage niche to generate a pro‐regenerative output [145]. In congestive liver disease, mechanical stress is selectively interpreted in pericentral liver sinusoidal endothelial cells (LSECs) through an integrin αV‐YAP‐CTGF axis that promotes a fibrogenic program [146]. These studies suggest that Hippo‐YAP/TAZ signaling does not produce a uniform output but instead leads to context‐dependent effects influenced by local cellular conditions and the microenvironment. Future studies will need to define more precisely how tissue environment and cellular identity impact Hippo‐YAP/TAZ signaling in various disease contexts. However, even with these emerging views of context‐dependent Hippo signaling, several key questions remain unanswered. How Hippo signaling prioritizes or integrates multiple systemic inputs within tissues across physiological contexts, and the identity of the molecular mediators that transmit Hippo‐dependent signals to distant organs, remain unclear. Furthermore, although accumulating evidence has linked Hippo signaling to metabolic disorders, cancer, and cardiovascular pathology, the causal relationships connecting organ‐specific Hippo activity to systemic disease progression are not yet fully resolved. Addressing these gaps will be essential to establishing Hippo signaling as an integrative framework for interorgan communication and to identify new therapeutic opportunities at the intersection of systemic physiology and disease.

## Author contributions

EJ and GS wrote the manuscript. GS and CL prepared the figures. GS, CL, and EJ edited the manuscript.

## Conflicts of interest

The authors declare no conflict of interest.
